# Intention to quit and its correlates among dieticians residing in the United Arab Emirates during the COVID-19 pandemic: A cross-sectional survey

**DOI:** 10.1371/journal.pone.0295904

**Published:** 2024-01-02

**Authors:** Farah Naja, Haydar Hassan, Hadia Radwan, Fares Kellany, Leila Cheikh Ismail, Mona Hashim, Wafa Helmi Rida, Salma Abu Qiyas, Mohamad Alameddine

**Affiliations:** 1 Department of Clinical Nutrition and Dietetics, College of Health Sciences, University of Sharjah, Sharjah, United Arab Emirates; 2 Research Institute of Medical and Health Sciences, University of Sharjah, Sharjah, United Arab Emirates; 3 College of Medicine, University of Sharjah, Sharjah, United Arab Emirates; 4 Public Health and Prevention Department, Dubai Health Authority, Dubai, United Arab Emirates; 5 Department of Health Care Management, College of Health Sciences, University of Sharjah, Sharjah, United Arab Emirates; University of Petra (UOP), JORDAN

## Abstract

**Background:**

The COVID-19 pandemic precipitated increased workload, stress, and burnout on healthcare providers on the frontlines of the pandemic, dieticians were no exception. Such unprecedented occupational risks and stressors contributed to a higher intention to quit, potentially leading to workforce shortages, and hindering the delivery of quality care, especially for patients with chronic conditions. The aim of this study was to examine the prevalence factors associated with the intention to quit among dieticians in the United Arab Emirates during a public health emergency.

**Methods:**

The study utilized a cross-sectional design with an online survey sent to dieticians between January and May 2021. The final version of the questionnaire included four sections: A sociodemographic section, intention to quit, work-related practices and challenges, as well as the resilience scale using the 25 items- Connor-Davidson Resilience Scale© (CD-RISC). Descriptive statistics as well as simple and multiple logistic regression analyses were carried out to explore factors associated with the intention to quit among dieticians.

**Results:**

Study results revealed that a quarter of dieticians intend to quit their jobs. Higher odds of intention to quit among dieticians were significantly associated with male gender, younger age, having a chronic condition, being non-resilient, feeling unappreciated, using online platforms for dietary counseling, reporting increased workload, and working from home or in a blended format during the pandemic.

**Conclusion:**

This study revealed a high intention to quit among dieticians during the COVID-19 pandemic and identified a few correlates for the intention to quit that could support the development of evidence-based interventions. Such interventions should address through targeted programs the challenges faced by male dieticians, younger dieticians, as well as dieticians with Chronic health conditions. Furthermore, the findings of this study showed that promoting resilience among dieticians is crucial in reducing their intention to quit.

## Introduction

Placed at the frontline in the battle against the COVID-19 pandemic, many healthcare professionals have faced unprecedented challenges and hardships. Physically, they have been exposed to a higher risk of infection due to close contact with COVID-19 patients, often working long hours in demanding conditions while wearing personal protective equipment [1, 2]. This increased risk has impacted both their well-being and that of their families, with many healthcare workers contracting the virus themselves and experiencing severe illness or even loss of life [3]. Moreover, on the mental and emotional front, healthcare workers witnessed the suffering and death of patients on a scale never seen before, leading to high levels of stress, anxiety, and trauma [4]. The constant pressure, fear, and uncertainty surrounding the pandemic have contributed to exhaustion and burnout among healthcare professionals [5]. According to the World Health Organization (WHO), during the COVID-19 pandemic, 23–46% of healthcare workers reported symptoms of anxiety, and 20–37% experienced symptoms of depression [6]. This heightened pressure, coupled with the relentless demands of the pandemic, led to an increase in intentions to quit among healthcare professionals, as they grapple with the physical, emotional, and psychological toll of the crisis.

Among healthcare professionals, dieticians were particularly at risk during the COVID-19 pandemic for several reasons. Dieticians, most often work in healthcare settings such as hospitals and clinics, where they interact closely with patients who may be infected with the virus. This proximity increases their exposure to the risk of contracting COVID-19 [7]. In the hospitals, many COVID-19 patients who were ill or recovering from illnesses required specialized dietary guidance and support to manage their health conditions, particularly those with comorbidities or compromised immune systems. This increased demand for the dieticians’ services placed dieticians in frequent contact with individuals who were potentially infected, further heightening their risk of exposure to the virus [8]. Dieticians’ medical services often require counseling, hence closer and longer contact with patients while wearing personal protective equipment, practicing social distancing, and implementing enhanced hygiene protocols. While these measures are essential for reducing the spread of the virus, they can also pose challenges and increase the workload for dieticians [9]. Furthermore, the COVID-19 pandemic has resulted in significant disruptions to healthcare systems, including staffing shortages and increased workloads. Dieticians may have had to take on additional responsibilities or work longer hours to meet the increased demand for their services. This increased workload, coupled with the stress and pressure of working in a high-risk environment, can contribute to fatigue and potentially compromise their immune systems, making them more susceptible to infections [10]. Forced to endure all the above-mentioned physical and psychological stresses that their work has imposed on them due to the pandemic, many dieticians are exposed to a high level of burnout, compromising their services as well as their well-being [11].

In the United Arab Emirates (UAE), during the COVID-19 pandemic, dietetic services were of utmost importance, especially considering the prevalent obesity and obesity-related diseases in the country, including diabetes and hypertension. According to a recent cross-sectional survey of 2142 adults living in Dubai, the prevalence of obesity among Emirati nationals had reached an alarming 39.6% [12]. Furthermore, the prevalence of diabetes among Emirati nationals ranges from 21% in males to 23% in females [13]. A recent systematic review and meta-analysis revealed that three out of ten adults in the UAE suffer from hypertension [14]. Given that obesity and these associated diseases are known risk factors for severe COVID-19 outcomes and associated mortality [15], the role of dieticians is critical in tailoring nutritional guidance and support to patients, helping them make informed choices to improve their overall health and strengthen their immune systems [16].

The increased workload, stress, and burnout experienced by dieticians on the frontlines of the pandemic can contribute to a higher intention to quit, potentially leading to workforce shortages and hindering the delivery of quality care, especially for patients with chronic conditions. The aim of this study is to examine the prevalence of intention to quit among dieticians in the UAE and identify the main sociodemographic characteristics and work-related practices and challenges associated with a higher intention to quit. Examining the intention to quit among dieticians during the COVID-19 pandemic is, therefore, essential for healthcare organizations to develop targeted interventions that support systems to improve job satisfaction, ensure the continuity of high-quality nutritional care, and effectively manage the long-term consequences of the pandemic.

## Methods

### Study design and target population

To investigate the intention to quit and its correlates among working dieticians residing in the UAE during the COVID-19 pandemic, a cross-sectional web-based online survey was carried out between January and May 2021. The questionnaire link was distributed to the dieticians via emails and mobile messages. The list of email addresses and phone numbers was obtained from the database of the Ministry of Health and Prevention (MOHAP) and Dubai Health Authority (DHA), as well as from various dietician associations in the country. The lists of dieticians obtained from these different sources were compared to avoid duplication of the contacts. In order to maximize the number of respondents, we opted not to sample from these lists. As such, all dieticians whose names and addresses were featured on these lists were sent the invitation to participate.

The inclusion criteria used in this study were as follows:1-Dieticians who are currently working in UAE; 2- Conversant in English or Arabic language. Dieticians were excluded if they were not currently working in the UAE. The online survey consisted of a link to an internet-based questionnaire on Google Forms with closed-ended questions in English and Arabic. Before completing the online survey, respondents were presented with an information form that described the purpose of the study, the procedure, the time needed to complete the questionnaire, as well as the voluntary nature of participation. A written informed consent form was obtained electronically from participants before enrollment in the study. Recruitment of participants started on February 1^st^ and ended on May 23^rd^, 2021.

The study protocol and questionnaire were reviewed and approved by the Research and Ethics Committee at the University of Sharjah (REC-20-12-07-01). The anonymity of the respondents was guaranteed during the data collection process. Sample size calculation was performed using Raosoft software [17]. Assuming a confidence level of 95%, a standard error of 5%, and a 50% probability of the outcome (intention to quit job), a total of 370 respondents were needed [18].

### Questionnaire used in data collection

A questionnaire with close-ended questions was developed following a thorough review of the literature [19–24]. A group of experts, including a nutrition epidemiologist, two clinical dietitians practicing in the UAE, and a health policy expert reviewed the developed questionnaire for both its content and face validity. For the content validity, the panel ensured that the questionnaire represented all the possible elements that ought to be considered to test the objective of this study. As for the face validity, the questionnaire was deemed by all panel members as appropriate to address the main elements needed to examine the study objectives. The questionnaire, originally in English and translated to Arabic and then back-translated to English. An expert who is bilingual and whose native language is Arabic translated into and from Arabic. It was then back-translated into English by a bilingual expert whose native language is English. The translated and back-translated versions of the questionnaire were compared, and any inconsistencies were cleared out to ensure accurate translation. Both versions of the questionnaire were pilot tested among 10 dieticians for clarity and simplicity and logical flow of the questions. Necessary modifications were made to the final version of the questionnaire considering the feedback received during pilot testing.

The final version of the questionnaire addressed the sociodemographic information of respondents, their intention to quit in addition to work-related practices and challenges. Sociodemographic characteristics included age, gender, marital status, the highest level of education attained, years of experience, place of work, and whether the respondent has any chronic health conditions. The intention to quit of the dieticians during the pandemic was examined using a Yes/No question: “Did you consider quitting your job during the COVID-19 pandemic?”. The remaining questions referred to practice-related characteristics of the respondents during the pandemic. It included Yes/No questions such as: using online platforms during COVID-19, being concerned about one’s health, and if counseled COVID-19 patients. In addition to the aforementioned questions, dieticians were asked if they were feeling appreciated as practicing dieticians during the pandemic with 5-point Likert scale answers: very appreciated, appreciated, neutral, unappreciated, and very unappreciated. Other questions were related to working conditions during COVID-19 questions: if they worked from the office/ home or blended type of working as well as asking if the workload was increased/decreased during the pandemic. Moreover, to examine the main challenges facing the dieticians during pandemic, the following question was addressed to the dieticians: “Which of the below do you perceive as a challenge during COVID-19?” and the respondents had the choice to select more than one answer from the following choices: Job satisfaction, job security, face-to-face counseling, work-life balance. The resilience of dieticians was examined using the 25 items-Arabic version of the Connor-Davidson Resilience Scale© (CD-RISC), each rated on a 5 point-Likert scale ranging from not true at all to true nearly all the time. The total score ranged between 0 and 100, with higher scores indicating better resilience [25].

### Statistical analysis

Frequencies and percentages were used to describe the characteristics of the study population.

Simple and multiple logistic regression analyses were performed to investigate the factors associated with the intention to quit among dietitians. The intention to quit, categorized as either "Yes" or "No," served as the dependent variable, while various characteristics of the respondents were examined as independent variables. In the initial phase of the analysis, a series of simple logistic regression models were applied to assess the association between each independent variable and the dependent variable, the intention to quit. Factors such as age, gender, and other variables were individually tested.

Subsequently, a multiple logistic regression model was constructed to examine the combined influence selected independent variables on the intention to quit. To select the variables that ought to be included in the multiple regression, the results of the simple regression were examined. Variables were selected if their p-values, reflecting the strength of their association with the intention to quit, were equal to or less than 0.2 in these simple regression analyses. Age and gender were included in the multiple regression model. Results of the logistic regression analyses were expressed as odds ratio (OR) with 95% Confidence Intervals (CI). A p-value less than 0.05 was considered statistically significant. All data analyses were carried out using the IBM SPSS statistics software (IBM Corp (2017). IBM SPSS Statistics for Windows, Version 25.0. Armonk, NY: IBM Corp).

## Results

A total of 371 dieticians consented to participate and responded to the questionnaire. The prevalence of intention to quit was 24.5% in the study population. Table 1 demonstrates the sociodemographic and practice-related characteristics of the respondents and their associations with intention to quit. Most of the respondents were females (87.6%; 325/371). The mean age of respondents was 34.47 ± 8.70. Most of the respondents indicated that they are currently married or had been ever married (62%; 230/371), have children (53.9%; 200/371), and have a Bachelor of Science degree as their highest level of education (65.2%; 242/371). Having a chronic health condition was reported by 27% of the respondents (101/371). One-third of respondents who reported having a chronic condition indicated an intention to quit their jobs (33.7%; 34/101), compared to those who did not report a chronic health condition (21.1%; 57/270 ‐ p<0.01). The respondents who had more than five years of experience (61.7%; 213/371) reported lower intention to quit their jobs compared with those with less than five years of experience (21.1% and 29.5%; respectively ‐ p = 0.05). Analysis of the responding dieticians’ resilience scores revealed that 52% were resilient and 48% were not resilient. The prevalence of intention to quit among the resilient group was lower (18.5%; 33/178), compared with the intention to quit among the non-resilient group (30.1%; 58/193 -p = 0.07). About two-thirds (62.3%; 231/371) of the respondents felt appreciated, out of whom 19.9% (46/231) indicated intention to quit job. On the other hand, 50% of those who felt unappreciated indicated the intention to quit job (p = 0.002). About half the respondents (51.5%; 191/371) reported a decrease in their workload during the COVID-19 pandemic and 26.1% (97/371) reported an increase in their workload during the same period. The intention to quit was significantly lower among dieticians who indicated no change in their workload during COVID-19 (16.9%; 14/83), compared with those reporting an increased workload (33%; 32/97 ‐ p = 0.04). The majority of responding dieticians indicated continuing to work at their workplace during the pandemic (68.7%; 255/371), while 31.3% indicated working from home or having blended work arrangements during the pandemic (31.3%; 116/371). Dieticians continuing to work at their workplace during the COVID-19 period had a significantly lower intention to quit job compared with those who worked from home or had a blended work arrangement (21.2% and 31.9%; respectively ‐ p = 0.019). The vast majority (91.1%) of the respondents were concerned about their health out of whom one-fourth expressed an intention to quit job (25.8%; 88/341). Conversely, those with no concern about their health had significantly lower intentions to quit their job (10%; 3/30 ‐ p = 0.036). Two-thirds (65.8%; 244/371) of the respondents had counseled COVID-19 patients with no significant difference in the intention to quit across this variable.

**Table 1:** Descriptive characteristics of study respondents (n = 371).

		Total	Intention to quit		P-value
			No n(%)	Yes n(%)	
**Sociodemographic characteristics**					
Age (years)		34.47±8.70	35.15 ±8.92	32.31 ± 7.6	**0.004**
Gender					
	Female	325 (87.6)	248 (76.3)	77 (23.7)	0.20
	Male	46 (12.4)	32 (69.6)	14 (30.4)	
Marital status					
	Ever married	230 (62)	176 (76.5)	54 (23.5)	0.32
	Never married	141(38)	104 (73.8)	37 (26.2)	
Do you have children?					
	No	171 (46.1)	125 (73.1)	46 (26.9)	0.20
	Yes	200 (53.9)	155 (77.5)	45 (22.5)	
Highest education					
	BSc	242 (65.2)	179 (74)	63 (26)	0.21
	Masters/ PhD	129 (34.8)	101 (78.3)	28 (21.7)	
Having any chronic health condition					
	No	270 (72.8)	213 (78.9)	57 (21.1)	**0.01**
	Yes	101 (27.2)	67 (66.3)	34 (33.7)	
**Practice-related characteristics**					
Years of experience					
	Less than 5	132 (38.3)	93 (70.5)	39 (29.5)	**0.05**
	More than 5	213 (61.7)	168 (78.9)	45 (21.1)	
Where do you practice?					
	Hospital-based	208 (56.1)	155 (74.5)	53 (25.5)	0.36
	Clinic (public and private)	163 (43.9)	125 (76.7)	38 (23.3)	
Resilience score					
	Resilient	193 (52)	135 (69.9)	58 (30.1)	**0.07**
	Not resilient	178 (48)	145 (81.5)	33 (18.5)	
Feeling of appreciation					
	Appreciated	231 (62.3)	185 (80.1)	46 (19.9)	**0.002**
	Neutral	116 (31.3)	83 (71.6)	33 (28.4)	
	Unappreciated	24 (6.5)	12 (50)	12 (50)	
Workload during COVID-19					
	Same	83 (22.4)	69 (83.1)	14 (16.9)	**0.04**
	Decreased	191 (51.5)	146 (76.4)	45 (23.6)	
	Increased	97 (26.1)	65 (67)	32 (33)	
Using online platforms during COVID-19					
	No	125 (33.7)	103 (82.4)	22 (17.6)	**0.017**
	Yes	246 (66.3)	177 (72)	69 (28)	
Working conditions during COVID-19					
	Home/blended	116 (31.3)	79 (68.1)	37 (31.9)	**0.019**
	Workplace	255 (68.7)	201 (78.8)	54 (21.2)	
Concerned about one’s health					
	No	30 (8.1)	27 (90)	3 (10)	**0.036**
	Yes	341 (91.9)	253 (74.2)	88 (25.8)	
Counseled COVID-19 patients					
	No	127 (34.2)	99 (78)	28 (30.8)	0.251
	Yes	244 (65.8)	181 (74.2)	63 (25.8)	

**Fig 1:**
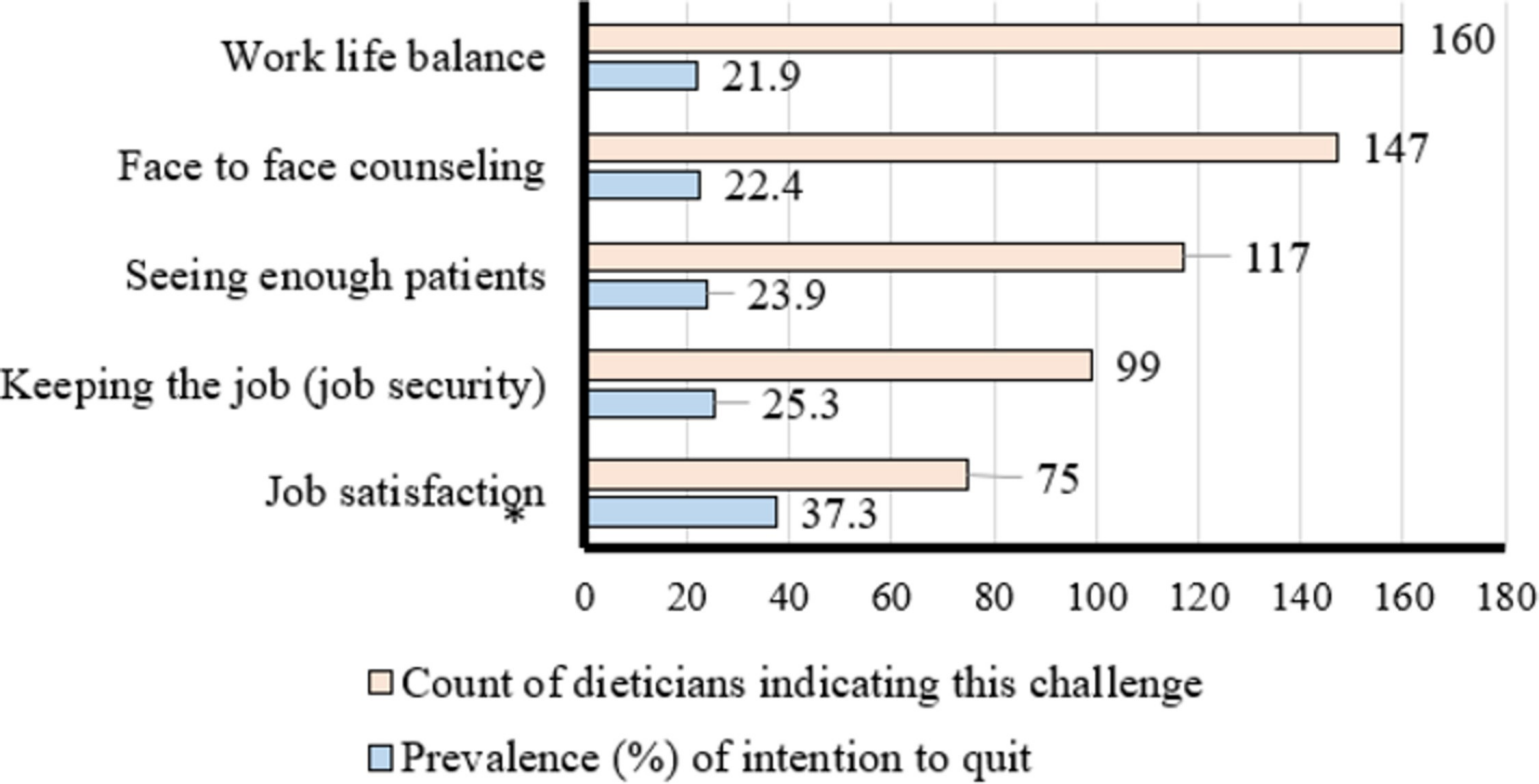
Prevalence of intention to quit according to challenges faced among dieticians during the COVID-19 pandemic (n = 371). * (p<0.05).

In regards to main job challenges during the pandemic, and as displayed in Fig 1, maintaining work-life balance was the top reported challenge (43.1%; 160/371) followed by the ability to offer face-to-face counseling, seeing enough patients, maintaining job security and job satisfaction (39.6% [147/371], 31.5%[117/371], 26.6%[99/371 and 20.2%[75/371]; respectively). However, it is noteworthy that the highest prevalence of intention to quit was among respondents who indicated challenges with their job satisfaction as compared to those who did not (p<0.05).

Table 2 illustrates the regression analysis using the intention to quit as a dependent variable. Male respondents had higher odds of intention to quit compared to their female counterparts (OR = 2.34; 95%CI: 1.06–5.40). Regarding age, older respondents had lower odds of intention to quit compared to younger respondents, and those with chronic health conditions had more than double the odds of intention to quit compared to those without a chronic condition (OR = 1.91; 95%CI: 1.06–3.46).

**Table 2:** Simple and multiple logistic regressions for the association of sociodemographic and practice-related characteristics with intention to quit (n = 371).

		Intention to quit					
		Simple logistic regression			Multiple logistic regression^*^		
		OR	p-value	95%CI	Adjusted OR^**^	p-value	95%CI
**Sociodemographic characteristics**		Simple Logistic Regression			Multiple Logistic Regression		
Age (years)		**.96**	**.008**	**.93-.99**	**.96**	**.049**	**.92–1.00**
Gender	Female	REF			REF		
	Male	**1.41**	.322	0.71–2.78	**2.34**	**.046**	**1.06–5.40**
Marital status	Never married	REF					
	Ever married	.86	.548	0.53–1.40	-		-
Having children	No	REF					
	Yes	0.80	.327	0.49–1.27	-		-
Highest education	BSc	REF					
	Masters/ PhD	0.79	.357	0.47–1.31	-		-
Have a chronic condition	No	REF			REF		
	Yes	**1.90**	**.013**	**1.14–3.14**	**1.91**	**.032**	**1.06–3.46**
**Practice-related characteristics**							
Years of experience	Less than 5	REF			REF		
	More than 5	0.64	.078	0.39–1.05	.99	.98	0.52–1.91
Practice location	Hospital based	REF					
	Clinic (public and private)	0.89	.630	0.55–1.43	-		-
Resilience^§^	Not resilient	REF			REF		
	Resilient	**0.53**	**.011**	**0.32–0.86**	**.45**	**.006**	**0.25–0.79**
Feeling of appreciation	Appreciated	REF			REF		
	Neutral	1.60	.075	0.95–2.68	1.67	.115	0.88–3.16
	Unappreciated	**4.02**	**.002**	**1.70–9.53**	**3.04**	**.030**	**1.11–8.32**
Workload during COVID-19	Same	REF			REF		
	Decreased	1.52	.218	0.78–2.95	1.07	.870	.49–2.34
	Increased	**2.43**	**.015**	**1.19–4.95**	**2.60**	**.023**	**1.14–5.94**
Using online platforms	No	REF			REF		
	Yes	**1.82**	**.028**	**1.07–3.12**	**2.34**	**.014**	**1.18–4.62**
Working modality	Home/blended	REF			REF		
	Workplace	**0.57**	**.027**	**0.35–0.94**	**0.50**	**.023**	**0.27–0.91**
Concern about one’s health	No	REF			REF		
	Yes	3.13	.066	0.92–10.57	1.69	.428	0.46–6.16
Counseled COVID-19 patients	No	REF					
	Yes	1.23	.423	0.74–2.05	-		-

(REF) indicates reference category in the logistic regression; Significant OR (˂0.05) are bolded; (-) indicated that the variable was not included in the multiple logistic regression model

^*^ Variables were entered in the multiple logistic regression model if, in the simple logistic regression, the resulting p-value of

^**^In this table the adjusted OR, resulted from the multiple logistic regression analysis

their association with the intention to quit was <0.2

§Resilient: above the median score (72); not resilient: below the median score (72)

Regarding practice-related characteristics, the respondents who were resilient and those who continued to work at their workplace during the pandemic had significantly lower odds of intention to quit (OR = 0.45; 95%CI: 0.25–0.79 and OR = 0.50; 95%CI: 0.27–0.91 respectively; p<0.05). Whereas those who felt unappreciated (OR = 4.02; 95%CI: 1.70–9.53, p<0.05), those who had an increased workload (OR = 2.60; 95%CI: 1.14–5.94, p<0.05) and those who used online platforms during COVID-19 pandemic (OR = 2.34; 95%CI: 1.18–4.62, p<0.05) showed significantly higher odds of intention to quit job (Table 2).

## Discussion

This study presents a rare attempt to examine the working conditions and the factors associated with the intention to quit among dieticians in the UAE. Most literature globally has classically focused on physicians and nurses and little attention was dedicated to critically reflecting on the working conditions and factors associated with intention to quit among dieticians. This is despite them being integral and valuable members of the healthcare delivery team, especially during public health emergencies and pandemics. The study showed that one in four dieticians intend to quit their jobs. The rates of intention to quit were significantly higher among dieticians who indicated ‘job satisfaction’ as a main challenge during the COVID-19 pandemic, as compared to those who did not. Higher odds of intention to quit among dieticians were significantly associated with male gender, younger age, having a chronic condition, not being resilient, feeling unappreciated, using online platforms for dietary counseling, reporting increased workload, and working from home or in a blended format during the pandemic.

Study findings reflect a destabilized workforce, with one in four dieticians expressing an intention to quit their jobs. The dearth of studies examining the intention to quit among dieticians during the COVID-19 pandemic did not support a comparison with the findings of this study. This underscores the importance of researchers including dieticians in future studies examining job satisfaction and intention to quit. Having said that, the intention to quit among dieticians is relatively lower compared to other front liners in the healthcare team. For example, a recent systematic review and meta-analysis on nurses’ intention to quit during the pandemic concluded that the estimated overall intention to quit among nurses was 31.7%. Nevertheless, the intention to quit among UAE dieticians remains disconcerting and calls for interventions aiming at enhancing their job satisfaction and retention. Such interventions should identify the root causes of job dissatisfaction among dieticians and the means to address them to ensure enhanced job satisfaction and retention.

Study results show that males had higher odds of expressing an intention to quit compared with their female counterparts. This could be attributed to multiple factors including the higher propensity of male dieticians to work in hospitals or long-term care facilities where they experience higher workloads and increased levels of stress and burnout. Another possibility could be the availability of fewer advancement opportunities for male dieticians in a female-dominated profession, especially in certain specialties such as maternal and pediatric nutrition. Lastly, male dieticians are reported to have increased difficulty in maintaining a work-life balance. This would be particularly relevant at times of public health emergencies and pandemics [26]. Retention programs for dieticians need to take note of the vulnerabilities of male dieticians who are more likely to quit job compared to their female counterparts.

The relatively younger dieticians have a significantly higher intention to quit compared with their older and more experienced counterparts. This could be attributed to multiple factors including the relatively older dieticians having gained experience in maneuvering difficult situations and mobilizing resources to support them at difficult times [27]. Older dieticians are more likely to be married with family responsibilities which makes them relatively less mobile [28]. Younger dieticians may be less able to practice independently and to swiftly adapt to the significant practice culture changes during a public health emergency without strong support from their institutions, their management, and their more senior colleagues [29]. Younger dieticians would need targeted support programs, especially during public health emergencies and protracted crises to decrease the propensity of their dissatisfaction, burnout, and turnover.

The odds of quitting job for dieticians reporting having a chronic condition are significantly higher compared with those who do not report a chronic illness. This is related to the fact that healthcare workers with chronic conditions are more likely to experience significant complications should they contract the virus, including higher odds of requiring placement on a ventilator or mortality [30]. The protection of health workers with chronic conditions may necessitate that they get exempted from serving on the frontlines during a pandemic to preserve their well-being and safety.

Dieticians who were not resilient also had higher odds of expressing an intention to quit their job. This is likely related to the fact that being resilient acts as a protective factor from the negative effects of the pandemic including stress and burnout [31, 32]. The pandemic is a time of significant changes and difficult experiences that would drain the energy of healthcare providers unless they found a way to resort to internal and external resources that would keep them to continue practicing amid the difficult context [33]. Managers are strongly encouraged to periodically assess the resilience of their healthcare workers, including dieticians, and extend support to those who display low resilience with counseling and training programs to prevent their turnover.

Dieticians who expressed feeling unappreciated at work had four times the odds of expressing an intention to quit their jobs. Healthcare workers need to feel appreciated and supported by their organization to improve job satisfaction, and commitment, and reduce their intention to quit. Reducing turnover rates, necessitates employers to create a culture of recognition and appreciation in the workplace. This is particularly relevant during emergencies and crises. This is because, as confirmed by our study, the higher the workload, the higher risk of dieticians expressing intentions to quit job. While some variables affecting turnover intentions are outside the control of managers and organizations, institutionalizing a culture of appreciation and recognition is within the control of organizations and could make a big difference during difficult times should a culture of recognition and appreciation be institutionalized [34–37].

Lastly, an important nuance in this study is the significantly higher odds of intention to quit working from home and offering online counseling. This is indicative that dieticians appreciate working within institutional boundaries and perhaps more importantly appreciate having face-to-face contact with their patients. The subjective reporting of weight, height, and other nutrition metrics may be inaccurate and personal contact with patients is essential for successful dietary counseling.

This study is among very few investigations addressing the dietetic practice during the COVID-19 pandemic and the first in the Gulf countries to specifically examine the intention to quit among this workforce. That said, it is important to consider a few limitations of the study’s findings. The online data collection may have resulted in a self-selection bias, hence affecting the generalizability of the results. However, during the pandemic, face-to-face data collection was not allowed for safety purposes. Furthermore, the online survey used in this study could have led to a social desirability bias. In addition, the cross-sectional nature of the survey does not allow to draw any causality inferences.

In conclusion, the findings of this study revealed a prevalent intention to quit among dieticians practicing in the UAE during the COVID-19 pandemic and identified a few correlates for the intention to quit that could be used to develop evidence-based interventions. Such interventions should address the challenges faced by male dieticians, such as workload management, advancement opportunities, and work-life balance. Support programs for younger dieticians should be established to provide mentorship, guidance, and resources to navigate difficult situations and adapt to changing practice cultures. Moreover, healthcare institutions should prioritize the well-being and safety of dieticians with chronic conditions, considering exemptions from frontline duties during pandemics. Furthermore, the findings of this study showed that promoting resilience among dieticians is crucial in reducing the intention to quit. Managers should assess and support dieticians with low resilience through counseling and training programs, enabling them to cope with pandemic-related challenges and continue practicing effectively. Future research is needed to investigate the impact of specific interventions and support programs on the retention and job satisfaction of dieticians.

## Supporting information

S1 File Naja et al. intention to quit data file. (XLSX)
